# A Highly Efficient Bismuth Salts-Catalyzed Route for the Synthesis of α-Aminophosphonates

**DOI:** 10.3390/molecules15118205

**Published:** 2010-11-12

**Authors:** Antara Banik, Sahil Batta, Debasish Bandyopadhyay, Bimal K. Banik

**Affiliations:** Department of Chemistry, The University of Texas-Pan American, 1201 West University Drive, Edinburg, TX 78541, USA

**Keywords:** bismuth salts, catalysis, imines, phosphites, α-aminophosphonates

## Abstract

A convenient synthesis of different types of α-amino phosphonates via one-pot solvent-free three component reactions of aldehydes, amines and phosphites catalyzed by bismuth salts has been investigated. Bismuth triflate is found to be the most effective catalyst for this reaction.

## 1. Introduction

The synthesis of α-aminophosphonates has received the attention of organic chemists as they represent structural analogues of the important α-amino acids. Various uses of α-amino phosphonates as antimicrobial [1,2,3], antioxidant [2], antitumor [4,5,6], antiviral [7] and enzyme inhibitors [8,9,10] have been discovered. A number of synthetic methods for the construction of α-aminophosphonates have been reported [11] but the nucleophilic addition reaction of phosphites to imines is the most powerful and attractive method. In this context, some catalysts and procedures have been reported such as boric acid [12], silica sulfuric acid [13], magnesium perchlorate [14], titanium dioxide [15], antimony chloride [16], oxalic acid [17], sulfonic acid functionalized ionic liquid [18], hexanesulphonic acid sodium salt [19], zirconium (IV) compounds [20], trifluoroethanol [21], sodium dihydrogen phosphate [22], tetramethyl guanidine [1,3], microwave irradiation [2,7], iron(III) chloride [4] *etc.* In addition, some of the reactions [23,24,25] are performed in organic solvents.

**Scheme 1:**
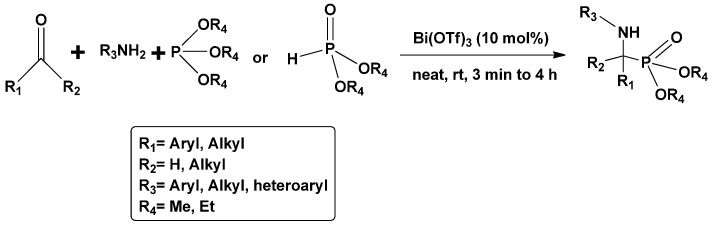
Synthesis of α-aminophosphonates via condensation of amines with carbonyl compounds and di/trialkyl phosphite in presence of bismuth salt as the catalyst.

Recently, bismuth salts have emerged as efficient Lewis acids due to their relatively low toxicity, ready availability at a low cost and tolerance of trace amounts of water. Therefore, we have investigated bismuth salts to address some of the limitations posed by known methods. Herein, in continuation of our research on bismuth salt-catalyzed reactions [26,27,28,29,30,31,32,33,34,35], we disclose a novel one-pot synthesis of structurally diverse α-aminophosphonates from aldehydes, amines and di/trialkyl phosphite (Scheme 1). It is also important to mention that we have reported the synthesis of several anticancer compounds using a bismuth salt-catalyzed reaction as the key step [36,37,38,39,40,41,42,43,44,45]. 

## 2. Results and Discussion 

Reaction of aldehydes with amines results in the formation of imine intermediates which subsequently reacts with di/trialkyl phosphites to produce the corresponding α-aminophosphonates (Scheme 1). A number of bismuth salts (10 mol%) have been screened using the reaction of benzaldehyde, aniline and trimethyl phosphite (equimolar ratio) as a probe. Bismuth triflate proved to be the ideal catalyst (Table 1).

Structurally diverse aldehydes, amines and phosphites were used in the presence of a catalytic amount (10 mol%) of bismuth triflate to afford the corresponding α-aminophosphonates in high to excellent yields (Table 2). Bhattacharya and Kaur reported [46] the synthesis of α-amino phosphonates using bismuth nitrate as the catalyst at room temperature and under microwave irradiation. A very high yield of the product was reported with various substrates. Based on our research on bismuth nitrate-catalyzed reactions [26,27,28,29,30,31,32,33,34,35], we are in a position to comment on this paper [46]. Bhattacharya and Kaur claimed to use bismuth nitrate pentahydrate as the catalyst. However, structure of this catalyst as written in this paper indicates that bismuth is monovalent. After careful search of the literature, monovalent bismuth nitrate was not available from any sources. In contrast to their paper, our results with trivalent bismuth nitrate pentahydrate produced α-aminophosphonates in comparatively low yield. Trivalent bismuth halides produced product in better yield than trivalent bismuth nitrate pentahydrate. A comparative study of the catalyst is shown in the Table 1. Aromatic aldehydes gave better yield probably because of the stability of the imines. Conjugated aldehyde produces product in lower yield. The reactions were compatible with different types of functional groups (Table 2). 

**Scheme 2:**
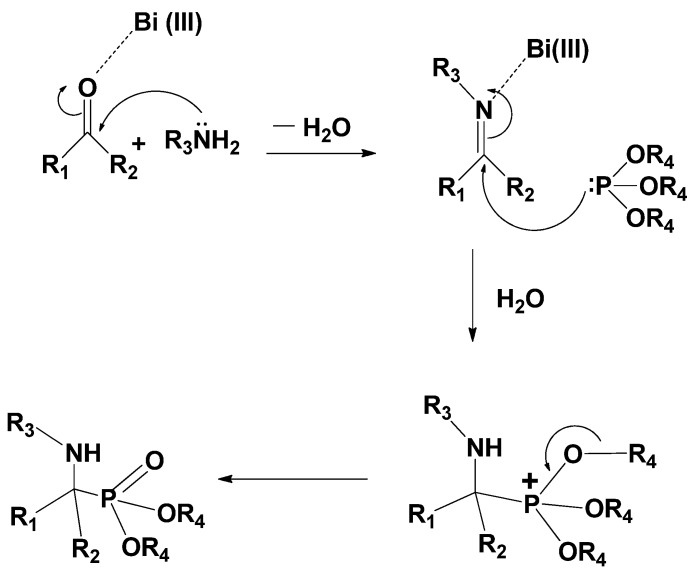
Plausible mechanistic pathway for the synthesis of α-aminophosphonates.

A plausible mechanism involves the formation of an imine by the addition of aldehyde and amine. It is believed that trivalent bismuth coordinates with the imine nitrogen to accelerate a nucleophilic reaction of phosphite to give a phosphonium intermediate, which then reacts with the water molecule formed during imine formation to yield the final product (Scheme 2). 

## 3. Experimental 

### 3.1. General 

Melting points were determined in a Fisher Scientific electrochemical Mel-Temp manual melting point apparatus (Model 1001) equipped with a 300 °C thermometer. FT-IR spectra were registered on a Bruker IFS 55 Equinox FTIR spectrophotometer as KBr discs. ^1^H-NMR (300 MHz) and ^13^C-NMR (75.4 MHz) spectra were obtained at room temperature with JEOL Eclipse-300 equipment using TMS as internal standard and CDCl_3_ as solvent. Analytical grade chemicals (Sigma-Aldrich incorporation) were used throughout the project. Deionized water was used for the preparation of all aqueous solutions.

*General procedure for the synthesis of α-aminophopsphonates*: Amine (1 mmol) and carbonyl compound (1 mmol) were mixed with di/trialkyl phosphite (1 mmol) in the presence of bismuth triflate (10 mol%). In the case of diamines (Entries 14 and 15, Table 2) the molar ratio of carbonyl compound and phosphite was double with respect to the diamine used. The reaction was monitored by TLC. After the completion of the reaction, dichloromethane (10 mL) was added to the reaction mixture and it was then washed successively with 5% NaHCO_3_ solution (2 mL) and brine (2 mL). The organic layer was dried with anhydrous sodium sulfate and concentrated. The products were found to be 95% pure from proton NMR study. Pure products were isolated through crystallization (dichloromethane-hexane). No column chromatography was needed for the purification of the products. Compounds obtained from the entries are reported. Our products have demonstrated satisfactory spectral and mp data compared with the reported values. 

## 4. Conclusions 

In conclusion, bismuth triflate was found to be an efficient catalyst in one-pot reaction of aldehydes, amines, and di/trialkyl phosphite to afford α-aminophosphonates. The main advantages of this method are mild conditions, clean, solvent-free reaction conditions and good to excellent yields. Application of this method toward the synthesis of biologically active molecules is under progress.

## Figures and Tables

**Table 1:** Bi salts-catalyzed one-pot synthesis of α-aminophosphonates. 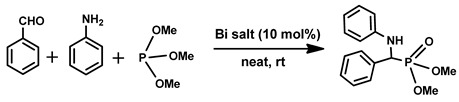

Entry	Bismuth salt (10 mol%)	Time (h)	Yield (%)
1	BiCl_3_	2	85
2	BiI_3_	5	75
3	Bi_5_O(OH)_9_(NO_3_)_3_	5	57
4	BiBr_3_	4	71
5	Bi(NO_3_)_3_.5H_2_O	5	67
6	Bi(OTf)_3_	3 min	98

**Table 2:** Synthesis of α-aminophosphonates via condensation of amines with carbonyl compounds and di/trialkyl phosphite in presence of bismuth triflate (10 mol%) as the catalyst following Scheme 1.

Entry	Carbonyl compound	Amine	Phosphite	Product	Time (min)	Yield (%)^a^	Ref
1	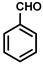	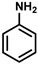	TMP^b^	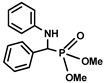	3	98	12
2	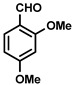	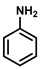	TEP^c^	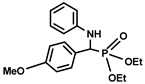	10	93	13
3	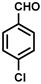	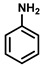	DEP^d^	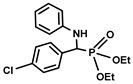	12	97	15
4	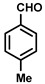	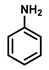	DEP	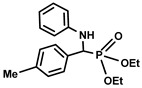	20	94	15
5	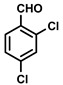	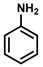	TMP	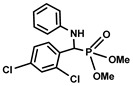	15	95	13
6	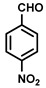	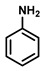	DEP	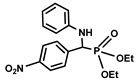	55	92	15
7	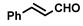	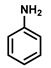	DEP	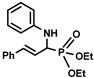	45	74	15
8	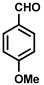	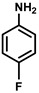	DEP	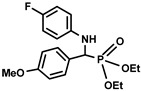	80	84	15
9	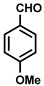	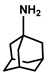	DMP^e^	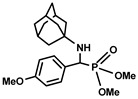	20	95	14
10	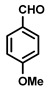	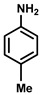	DEP	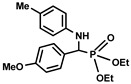	90	82	15
11	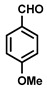	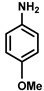	DEP	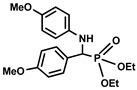	30	97	15
12	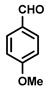	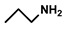	DEP	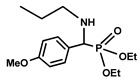	45	89	15
13	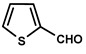	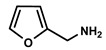	DMP	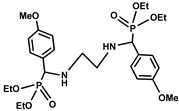	40	86	1
14	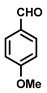	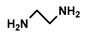	DEP	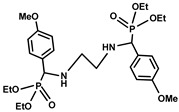	75	86	15
15	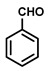	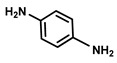	DEP	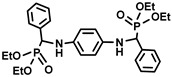	25	91	4
16	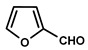	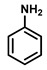	TMP	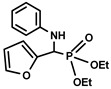	15	96	17
17	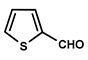	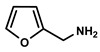	DEP	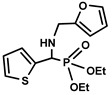	65	82	1
18	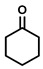	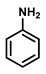	DEP	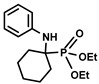	240	72	15

^a^ isolated yield; ^b^ trimethyl phosphite; ^c^ triethyl phosphite; ^d^ diethyl phosphite; ^e^ dimethyl phosphate.
